# What Is the Role for Pediatric Endocrinologists in the Management of Skeletal Dysplasias?

**DOI:** 10.1210/clinem/dgad726

**Published:** 2023-12-11

**Authors:** Nadia Merchant, Lynda E Polgreen, Ron G Rosenfeld

**Affiliations:** Department of Endocrinology and Diabetes, Children's National Hospital, Washington, DC 20010, USA; Department of Pediatrics, George Washington University School of Medicine and Health Sciences, Washington, DC 20037, USA; Department of Pediatrics, The Lundquist Institute for Biomedical Innovation at Harbor-UCLA Medical Center, Torrance, CA 90502, USA; Department of Pediatrics, Oregon Health & Science University, Portland, OR 97239, USA

**Keywords:** skeletal dysplasia, growth, targeted therapy, role

## Abstract

Children with skeletal dysplasias have not been consistently managed by pediatric endocrinologists despite the recognized expertise of these practitioners in managing genetic growth disorders. Growth-altering treatments have broadened the role of the pediatric endocrinologist to manage and sometimes become primary coordinators for genetic disorders such as Turner syndrome and Prader-Willi syndrome. We illustrate how recent advances in understanding the pathophysiology of skeletal disorders and the development of targeted treatments provide an opportunity for pediatric endocrinologists to further expand their role in managing certain skeletal dysplasias, including achondroplasia.

Historically, pediatric endocrinologists have treated children and adolescents with hormone-related conditions, such as diabetes, growth disorders, pubertal abnormalities, differences/disorders of sex development, obesity, bone and mineral disturbances, hypoglycemia, and other disorders relating to the adrenal, parathyroid, thyroid, and pituitary glands (1). Children with skeletal dysplasias have not been consistently managed by pediatric endocrinologists, despite the recognized expertise of these practitioners in managing genetic growth disorders (2). This is primarily due to the lack of an effective therapy for skeletal dysplasias. In this paper, we illustrate how growth-altering treatments have broadened the role of the pediatric endocrinologist in the past and how recent advances in understanding the pathophysiology of skeletal disorders and the development of targeted treatments provide an opportunity for pediatric endocrinologists to further expand their role in managing certain skeletal disorders.

## Looking Back: The Role of Pediatric Endocrinologists in the Treatment of Turner Syndrome and Prader-Willi Syndrome

### Turner Syndrome

Turner syndrome, a chromosomal disorder affecting approximately 25 to 50 per 100 000 live-born female infants, is associated with short stature, hypergonadotropic hypogonadism, infertility, gastrointestinal, hepatic, and autoimmune disorders, neurocognitive and psychosocial issues, ear and hearing issues, cardiovascular malformations, and increased risk of mortality (3-5). Historically, the care of children with Turner syndrome was managed by a variety of specialists, reflecting the wide range of disease complications. Even after growth failure and short stature became well-described central attributes of the disorder, the role of the pediatric endocrinologist was usually limited to treating referred adolescents for estrogen replacement therapy to induce puberty, maintain secondary sex characteristics, attain peak bone mass, and normalize uterine growth for possible future pregnancy (3, 6).

Although short stature in Turner syndrome is better attributed to a characteristic skeletal dysplasia than a hormonal deficiency, studies show that exogenous recombinant human growth hormone (rhGH) increased adult height in children with Turner syndrome. This led to Food and Drug Administration approval of rhGH in Turner syndrome in 1996 (4). In 2017, the International Turner Syndrome Consensus Group published clinical practice guidelines for the management of Turner syndrome throughout the lifespan and an accompanying patient/family-oriented summary of the guidelines (3, 7). The guidelines recommend starting rhGH therapy early (around 4-6 years of age) in children with Turner syndrome with low height velocity (3), which resulted in an increase in referrals. Pediatric endocrinologists are now often the primary coordinators of medical care for their patients with Turner syndrome, due to the need for frequent follow-up for rhGH management, and they play a crucial role in developing and expanding clinics specializing in comprehensive management of Turner syndrome.

### Prader-Willi Syndrome

Prader-Willi syndrome, caused by the lack of expression of several genes on the paternally derived chromosome 15, occurs in approximately 1 in 15 000 to 1 in 25 000 live births, with both male and female individuals affected (8). First described in 1958, Prader-Willi syndrome is characterized by severe infantile hypotonia, with early feeding problems and difficulty gaining weight, early-childhood hyperphagia, resulting in obesity and its attendant complications (eg, diabetes, obstructive sleep apnea), hypogonadism causing genital hypoplasia, incomplete pubertal development and usually infertility, characteristic facial features, developmental delay/mild intellectual disability, short stature, a distinctive behavioral phenotype, and death typically by the fourth decade of life (8-10).

For many decades, because there were no viable treatments for Prader-Willi syndrome and patients presented with a variety of disorders, treatment was scattered among various specialties. The etiology of growth failure in patients with Prader-Willi syndrome remains unclear, as some, but not all, have evidence of growth hormone insufficiency. In 2000, rhGH was approved for the treatment of growth failure in children with Prader-Willi syndrome. In addition to improving linear growth (11), rhGH in children with Prader-Willi syndrome also appears to improve body composition (11-13), bone mineral density (11), physical function (11), and may have positive effects on development and behavior (14). The availability of rhGH for Prader-Willi syndrome provided pediatric endocrinologists with an opportunity to expand their expertise in treating this complex disorder and encouraged them to begin serving as the patient's primary provider in certain centers (15).

## Looking Forward: Opportunities to Further Expand the Role of Pediatric Endocrinologists in Treating Skeletal Dysplasias

The incidence for skeletal dysplasias appears to be approximately 1 per 5000 live births (16). Although more than 450 skeletal dysplasias have been identified (2), we focus the discussion below on achondroplasia, the most common skeletal dysplasia and one for which potential therapies have emerged.

Achondroplasia, caused by autosomal dominant mutations in the fibroblast growth factor receptor 3 (*FGFR3)* gene, results in short stature and a variety of clinical manifestations that negatively impact health and quality of life throughout the lifespan (17). Because achondroplasia is a growth plate disorder affecting bone that lacked potential treatments until very recently, patients were treated by a variety of specialists and primarily followed by pediatricians, geneticists, otolaryngologists, neurosurgeons, and orthopedists, as needed for various medical and psychosocial complications (18).

A variety of drugs have been under investigation for achondroplasia to target the growth plate to stimulate chondrocyte differentiation and hypertrophy. These include analogues of C-type natriuretic peptide (CNP), FGFR3-selective tyrosine kinase inhibitors, anti-FGFR3 antibodies, aptamers targeting FGF2, and soluble forms of FGFR3.

Vosoritide became the first treatment for achondroplasia to be approved, in 2021 in the United States and Europe and in 2022 in Japan, Brazil, and Australia (19). Vosoritide is a biological analogue of CNP, a small peptide and potent stimulator of endochondral ossification. CNP binds to its receptor, natriuretic peptide receptor-B (gene *NPR2*) causing transformation of guanosine 5′-triphosphate (GTP) into cyclic guanosine monophosphate (cGMP). In the growth plate, CNP binds to NPR-B which leads to changes in gene expression in the chondrocytes, increasing proliferation and differentiation of chondrocytes and inhibiting the mitogen-activated protein kinase (MAPK) pathway (20). A phase 3, randomized, double-blind, placebo-controlled, multicenter trial of vosoritide (NCT03424018) showed that daily subcutaneous injections administered to children aged 5 to 18 years with achondroplasia over a 1-year period increased height velocity by an adjusted difference of 1.57 cm/year (*P* < .0001) and height Z-scores by an adjusted difference of 0.28 (*P* < .0001) (21). In the open-label phase 3 extension study, in which participants who had been assigned to active treatment continued to receive vosoritide for an additional 2 years compared with those who had been assigned to placebo and had 2 years of untreated measurements using data from a prior observational study (NCT01603095), there was a statistically significant greater decrease in upper-to-lower body segment ratio in vosoritide-treated vs untreated patients (difference in least squares mean change of −0.05 (95% CI −0.09, −0.01), a maintenance of improvement in annualized height velocity observed in the randomized placebo-controlled trial (5.52 cm/year at week 104 in the treated group), and a difference in least squares mean change (95% CI) in height Z-score favoring the treated group of +0.44 (0.25, 0.63) at week 104 (22). In addition, a completed phase 2, randomized, double-blind, placebo-controlled trial (NCT03583697) of vosoritide in infants and young children (aged 0 to <60 months) with achondroplasia, showed a height gain of 0.92 cm/year in vosoritide-treated vs placebo-treated children (23). Additional studies (NCT04554940) are being conducted to provide insight into whether earlier initiation of vosoritide yields greater height gains along with decreased complications, such as foramen magnum stenosis.

The CNP/NPR2 pathway is central to growth of the chondrocyte; therefore, patients with genetic causes of short stature that interact with this pathway could benefit from this treatment approach. Positive outcomes of vosoritide in achondroplasia have inspired trials of vosoritide in other genetic diseases causing short stature related to the CNP/NPR2 pathway (24). An Italian registry study of individuals with achondroplasia or hypochondroplasia (NCT05328050) and an investigator-initiated, open-label, single-arm study (NCT04219007) of the effects of vosoritide in patients with various skeletal dysplasias, including hypochondroplasia, *ACAN* variants, *SHOX* gene defects, Noonan syndrome, and neurofibromatosis type 1, are expected to provide preliminary information about the effectiveness of vosoritide for treatment of these other skeletal disorders.

There are other treatments in clinical development that target the FGFR3/CNP pathway for treatment of achondroplasia, including TransCon CNP (Ascendis Pharma, Copenhagen, Denmark) and infigratinib (Truseltiq; QED Therapeutics, Brisbane, CA) (25). TransCon CNP is a long-acting CNP compound designed to provide sustained systemic CNP release to the growth plate and will be administered by weekly subcutaneous injections (25). TransCon CNP has been shown to improve the skeletal phenotype in mice (26). A phase 1, randomized, placebo-controlled, single-ascending dose trial in 45 healthy adult males showed that TransCon CNP provided continuous systemic exposure to CNP over at least 7 days postdose and was well tolerated with no serious treatment-emergent adverse events or discontinuations (27). Two ongoing phase 2, multicenter, randomized, placebo-controlled, dose-escalation trials (NCT05246033, NCT04085523) are investigating the safety, efficacy, and pharmacokinetics of multiple subcutaneous doses of TransCon CNP administered once weekly in prepubertal children (aged 2-10 years) with achondroplasia.

Infigratinib is an oral monoclonal selective FGFR1-3 selective tyrosine kinase inhibitor in development for FGFR-related conditions, including achondroplasia, and has been shown to be effective in an achondroplasia mouse model (28). Infigratinib is being evaluated in PROPEL (NCT04035811) and PROPEL 2 (NCT04265651) (29). PROPEL is a prospective, noninterventional clinical study designed to characterize the natural history and collect baseline data of children with achondroplasia over 6 to 24 months (29). Children aged 3 to 11 years with achondroplasia who complete ≥6 months in PROPEL will be eligible for PROPEL 2, a phase 2, open-label study of infigratinib in children with achondroplasia consisting of a dose-escalation, dose-finding phase and a dose-expansion phase to confirm the selected dose and a pharmacokinetics substudy (29). The primary endpoints for PROPEL 2 are treatment-emergent adverse events, change from baseline in annualized height velocity, and pharmacokinetic parameters (29).

## New Therapies Offer an Opportunity to Expand the Role of Pediatric Endocrinologists in the Treatment of Achondroplasia and Other Skeletal Dysplasias

Just as the emergence of rhGH as an effective growth-altering treatment for Turner syndrome and Prader-Willi syndrome resulted in an increased need for pediatric endocrinologists to develop expertise in treating patients with these disorders, current and emerging therapeutics for skeletal disorders offer another opportunity to expand the role of pediatric endocrinologists. Pediatric endocrinologists, who have particular expertise in evaluating growth, are well suited to prescribe growth-altering treatments, to follow patients to assess growth and other indicators of health, and to serve as the medical home for patients with complex medical conditions. Importantly, pediatric endocrinologists have workflows for prescribing and obtaining prior authorizations for injectable medications and have support personnel who are skilled in teaching patients/families how to administer injections and in speaking with families and patients of different developmental ages (including puberty) to promote adherence to long-term injectable treatments.

It will be important for pediatric endocrinologists to manage patients’ and families’ expectations about the benefits of current and emerging treatments because complete knowledge about their efficacy is lacking. It is hoped that targeted treatments will mitigate the health comorbidities of achondroplasia and improve quality of life, goals that may be even more important to patients/families than increasing height. Hopefully, ongoing research will provide more information about the full benefits of vosoritide and investigational targeted treatments.

## Preparing and Supporting Pediatric Endocrinologists for an Expanded Role

Skeletal dysplasia clinics have traditionally been a multidisciplinary clinic with close partnership between expert geneticists, genetic counselors, orthopedics, neurosurgeons, otolaryngologists and the pediatricians and often other specialties including radiology and maternal fetal medicine. Pediatric endocrinologists who are prescribing treatments for achondroplasia in order to expand access to care with new emerging treatments may become the patient's consistent provider seen multiple times a year. Hence, pediatric endocrinologists need to be knowledgeable about developmental milestones, screening recommendations, treatment guidelines, resources for patients and families, and known complications in achondroplasia. Pediatric endocrinologists must work in partnership with skeletal dysplasia clinics, especially geneticists to ensure the best overall care since these patients have other complications that must be followed closely and to facilitate appropriate timely referrals.

In addition, pediatric endocrinologists will need to educate themselves on appropriate terminology (eg, dwarfism, little person) and avoid using terms that would be considered offensive (eg, when comparing height of the patient, the referent term *normal height* should be replaced by the term *average height*) (30). It is important to recognize that some families will decide not to pursue treatment and so providers must be capable of using patient-centered decision making when counseling families and patients (31).

The impending expansion of treatment options addressing the pathophysiology of achondroplasia offers an opportunity for interested pediatric endocrinologists to develop expertise in this area as well as other skeletal dysplasias and metabolic bone disorders with available growth-altering treatments. Endocrinology professional societies, academic programs, and pharmaceutical companies could all play important roles in helping to develop this larger cadre of pediatric endocrinologists. For example, professional societies could develop workshops and symposia on practices of excellence for pediatric endocrinologists interested in expanding their role in the management and treatment of skeletal disorders. Developing consensus-based clinical and accompanying lay guidelines for the treatment of specific skeletal growth disorders will be essential to expanding the pediatric endocrinologist's role in treating conditions with emerging growth-altering treatments, as has been seen with Turner syndrome (3, 7). Guidelines for pediatric endocrinologist fellowship training should include training in the management of children with skeletal dysplasias and should include education in the associated comorbidities. Pediatric endocrinology academic programs should encourage the development of faculty, especially younger faculty, who have an interest in developing expertise in the treatment of a fuller range of pediatric growth and skeletal.

We expect that some pediatric endocrinologists who develop expertise in management of skeletal dysplasias will want to serve as the “treatment home” for patients; they will want to focus on the treatment of growth failure and endocrinopathies but may not have all the tools to be responsible for coordinating patient care. A smaller number of pediatric endocrinologists will be able to both treat the disease manifestations within their purview of expertise and work with a strong partnership to provide multidisciplinary or intradisciplinary care (“medical home”). Developing region-specific networks of specialists who have expertise in treating specific rare skeletal disorders would be a valuable resource for these pediatric endocrinologists.

## Conclusion

The purview of pediatric endocrinologists has expanded with the development and emerging treatments of metabolic bone disorders and growth-altering treatments for skeletal dysplasia. The current and impending availability of rare disease drugs that impact growth and hopefully decrease other complications should spur excitement among pediatric endocrinologists interested in developing expertise in skeletal disorders. There is an urgent need for a concerted effort to ensure that adequate resources are available to screen and evaluate children with skeletal dysplasias in a timely manner, to refer appropriate children to clinicians with expertise in managing these rare conditions and their complications, and to develop models of care that will optimize care of these complex patients.

## Data Availability

Data sharing is not applicable to this article as no datasets were generated or analyzed during the current study.

## Abbreviations

CNP: C-type natriuretic peptide
FGFR: fibroblast growth factor receptor
NPR2: natriuretic peptide receptor-B
rhGH: recombinant human growth hormone
